# Scheelite weathering and tungsten (W) mobility in historical oxidic-sulfidic skarn tailings at Yxsjöberg, Sweden

**DOI:** 10.1007/s11356-019-07305-1

**Published:** 2019-12-21

**Authors:** Lina P.B. Hällström, Lena Alakangas, Olof Martinsson

**Affiliations:** 1 Applied Geochemistry, Luleå University of Technology, Luleå, Sweden; 2 Ore Geology, Luleå University of Technology, Luleå, Sweden

**Keywords:** Skarn tailings, Scheelite weathering, Tungsten mobility, Carbonate exchange, Hydrous ferric oxides, Goethite

## Abstract

More knowledge of the geochemical behavior of tungsten (W) and associated contamination risks is needed. Therefore, weathering of scheelite (CaWO_4_) and secondary sequestration and transport of W to groundwater in historical skarn tailings and surface water downstream of the tailings were studied. The tailings contained 920 mg/kg W, primarily in scheelite. Mineralogical and geochemical analyses were combined to elucidate the geochemical behavior of W in the tailings, and water samples were taken monthly during 2018 to monitor its mobility. In the tailings, a large peak of W was found at 1.5 m depth. There, 30 wt%. of W was present in easily reducible phases, indicating former scheelite weathering. Currently, W is being released from scheelite to water-soluble phases at 2.5 m depth. The release of WO_4_^2−^ is hypothetically attributed to anion exchange with CO_3_^2−^ released from calcite neutralizing acid produced from pyrrhotite oxidation in the upper tailings and transported downwards to pH conditions > 7. Higher concentrations of dissolved W were found in the groundwater and particulate W in downstream surface water than in reference water, but they were lower than current contamination thresholds. Tungsten showed correlations with hydrous ferric oxides (HFO) in both the tailings and surface water.

## Introduction

In recent decades, concerns regarding elevated concentrations of tungsten (W) in the terrestrial environment have been raised (Cui and Johannesson 2017; Datta et al. 2017; Koutsospyros et al. 2006; Lemus and Venezia 2015; Strigul 2010; Zoroddu et al. 2018, and references therein). High concentrations combined with poor knowledge of its geochemical behavior and toxicity has led to the US Environmental Protection Agency (2014) classifying W as an emerging contaminant of concern, and in Russia it was classified as a highly dangerous contaminant in aquatic systems in 2009 (Strigul et al. 2009). Tungsten has previously been considered an immobile metal and therefore has not been expected to have adverse environmental or toxicological effects (Strigul 2010). However, recently published reports describe elevated W concentrations in ground and surface water (Candeias et al. 2015; Gurbanov et al. 2015; Mohajerin et al. 2014; Johannesson et al. 2013; Seiler et al. 2005), bioaccumulation in higher trophic levels of ecosystems (Kennedy et al. 2012; Lin et al. 2014; Lindsay et al. 2017; Wilson and Pyatt 2006; Wilson and Pyatt 2009), and adverse effects on plants, fishes, and other organisms (Strigul et al. 2005; Strigul et al. 2010; Strigul 2010). The geochemical behavior of W is still not fully understood and research on its mobility is needed. What is known is that W belongs to group 6 of the periodic table, and has similar behavior to Mo (Gustafsson 2003; Kashiwabara et al. 2013; Kreissl et al. 2016). It forms monometric tungstate (WO_4_^2−^) in natural waters with near-neutral or alkaline pH and can form several polyoxyanionic species in acidic waters (Koutsospyros et al. 2006). Tungsten has high affinity for hydrous ferric oxides (HFO) at pH conditions below 8 (Gustafsson 2003; Kashiwabara et al. 2013; Kreissl et al. 2016) and its adsorption to and/or co-precipitation with ferrihydrite and goethite are believed to be strong scavenging processes in the environment (Cui and Johannesson 2017; Kashiwabara et al. 2013).

Elevated concentrations of W have been found downstream of W-rich ore deposits and mining areas (Candeias et al. 2015; Lin et al. 2014; Gurbanov et al. 2015; Petta et al. 2014; Seiler et al. 2005; Wilson and Pyatt 2006; Wilson and Pyatt 2009), but associated W releases have been poorly studied. More than 50% of W in primary minerals is found as scheelite in skarn ore deposits (Kwak 2012; Ray et al. 1995; Werner et al. 1998) and in historical times more than thirty scheelite skarn deposits have been mined at large scale (Kwak 2012; Meinert 1992; Werner et al. 1998). Historical tailings might pose higher risks of W contamination than active mines, due to careless disposal and high W contents resulting from inefficient extraction techniques (Gao et al. 2016; Hällström et al. 2018a; Marinakis and Kelsall 1987). Furthermore, release of W from scheelite may be promoted in skarn tailings by the high abundance of carbonates. Thus, several laboratory studies have detected release of W from scheelite in groundwater with near-neutral pH conditions, and hypothetically attributed it to anion exchange with CO_3_^2−^ (Atademir et al. 1979; Marinakis and Kelsall 1987; Montgomery and McKibben 2012). Accordingly, in historical skarn tailings at Yxsjöberg in Sweden, Hällström et al. (2018a, 2018b) found indications of scheelite weathering and W release. Moreover, monitoring by the local municipality detected W in groundwater, freshwater, and sediments as far as 5 km from the mining area (Höglund et al. 2004; Höglund et al. 2005). Thus, in this study, geochemical and mineralogical analyses were combined to seize the opportunity to study the weathering of scheelite, secondary sequestration of W in the tailings, and the transport of W to receiving groundwater and downstream surface waters at Yxsjöberg.

## Study area

**Fig. 1:**
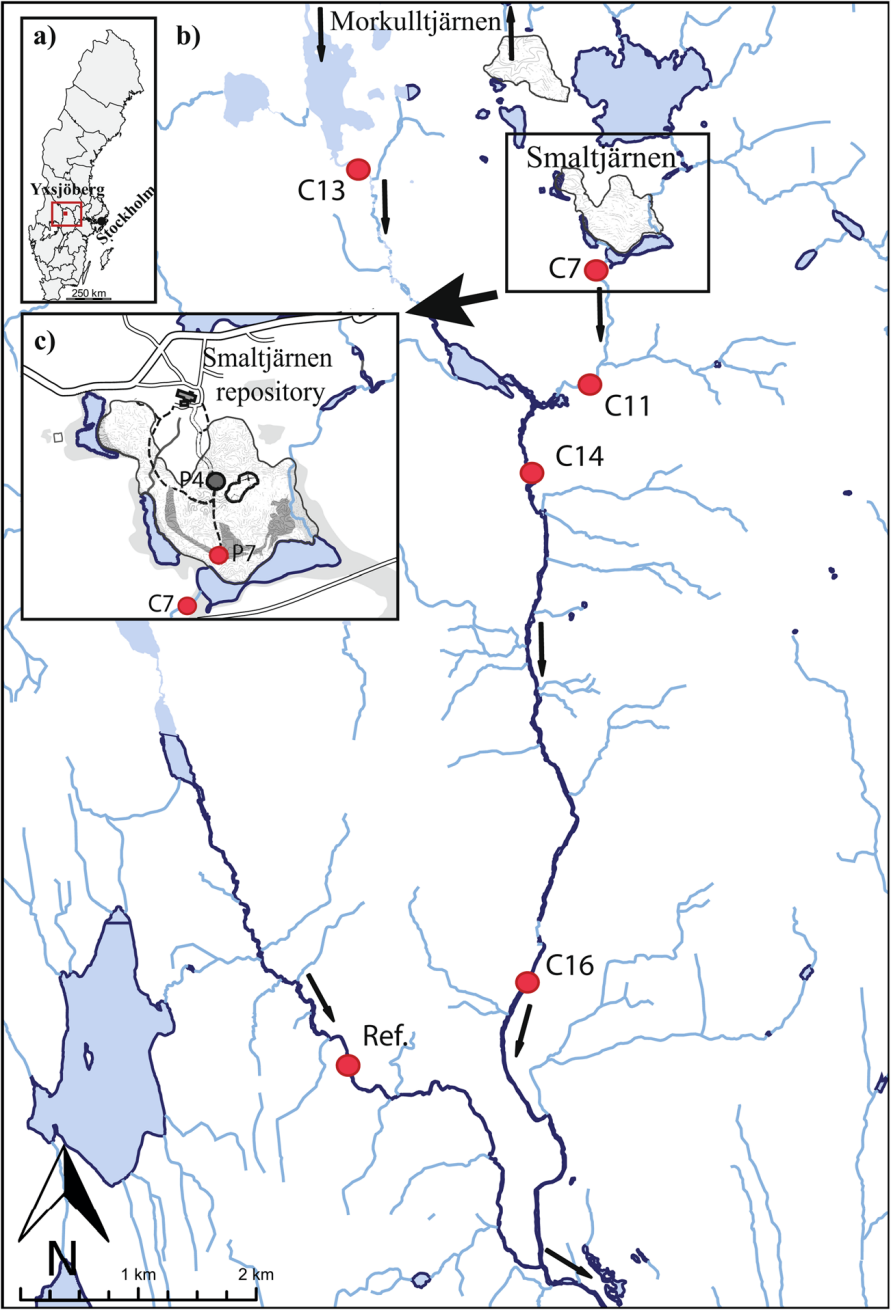
The map shows the localization of **a**) Yxsjöberg mining area in Sweden, **b**) the water samples (red dots) taken in the surface water downstream the two tailings repositories (Smaltjärnen and Morkulltjärnen) and the reference point, and **c**) the intact tailings core (grey dot: P4) and the groundwater samples (red dot: P7) in Smaltjärnen Repository. The arrows shows the water flow direction

In the Smaltjärnen repository, 2.8 million tons of tailings from the closed W, Cu, and fluorite mine at Yxsjöberg, Sweden (1918–1989) have been stored for more than 50 years. Detailed information about the site is presented by Hällström et al. (2018a). The tailings were deposited in the Smaltjärnen Repository during two active mining periods: 1918–1920 and 1935–1963 (Fig. 1). The Smaltjärnen repository area consisted of bogs and swamps, and no dams controlled the deposition of the tailings (Rothelius 1957). Lake Smaltjärnen is located directly south of the repository and people live in the vicinity of the tailings. In the repository, average contents of Be, Bi, Cu, Sn, W, Zn, F, and S in the tailings are 284, 495, 946, 559, 960, and 301 ppm and 1.9 and 1.2 wt%, respectively, according to analyses of 99 samples. Ca-rich silicates constitute 87.5 wt% of the tailings, and minerals including fluorite, calcite, pyrrhotite, pyrite, chalcopyrite, scheelite, bismuthinite, cassiterite, danalite, and magnetite are also abundant (Hällström et al. 2018a). Between 1969 and 1989, tailings were deposited in Morkulltjärnen Repository in the north (Fig. 1). Unlike Smaltjärnen, Morkulltjärnen is controlled by dams, covered by vegetation and partly saturated (Höglund et al. 2004).

## Materials and methods

### Field sampling

A representative core (P4) of four sampled by Hällström et al. (2018a) was chosen for a detailed study (Fig. 1). The intact vertical core reached a depth of 7.5 m and was taken in a Plexiglas tube (Ø 2″, 1.2 m each) and divided into 36 subsamples, 10–15 cm thick each. The samples were stored in cool and dark conditions in diffusion-tight bags, then analyzed by an accredited laboratory (ALS Minerals) for total concentrations of 66 elements. For detailed descriptions of the analytical methodology, standard reference materials, and detection limits, see Hällström et al. (2018a). As illustrated in Fig. 1, a groundwater pipe (P7) was installed in the tailings downstream of P4.

### Environmental mineralogy

Polished uncovered thin sections of 11 subsamples of P4 were examined, in duplicate, with transmitted and reflected light optical microscopy, using an Eclipse LV100POL instrument (Nikon). A modified alteration index (AI) method similar to those by Moncur et al. (2009) and Blowes and Jambor (1990) were used on the minerals in the thin sections of P4 to distinguish between oxidized and unoxidized environments in the tailings core. The AI classified unaltered pyrrhotite and calcite as 0 and completely altered/depleted minerals as 10. The extent of secondary formations of HFO was also taken into account as an indicator of an oxidized environment. The absences of HFO were classified as 0 and high abundance was classified as 10.

Mineral grains and rims of interest in the thin sections were examined by scanning electron microscopy (SEM) with energy dispersive X-ray spectroscopy (EDS) using a high-resolution Zeiss Merlin^TM^ FE-SEM (10 KeV and 1 μA) system and Aztec Software from Oxford Instruments. Raman vibrational spectroscopy was used on chosen thin sections from 3.6 m depth to determine the HFO around scheelite, pyrrhotite, and magnetite. Measurements with a green laser (532 nm) were acquired with a Bruker Raman Scope, Olympus BX51 microscope, and OPUS 7 Senterra software. Scheelite was measured with 10 mW, 5 s and 5 repetitions, and HFO around scheelite, magnetite, and pyrrhotite were measured with 0.2 mW, 1 s and 10 repetitions. Precautions using low laser power measurements were taken to avoid mineral transformation.

### 7-step sequential extraction

Five samples from P4 were sent to SGS Canada Inc. (Lakefield, ON, Canada) for 7-step sequential extraction according to Dold (2003). This procedure is intended to extract: (1) water-soluble phase, (2) exchangeable phase, (3) easily reducible minerals (e.g., oxyhydroxides), (4) resistant reducible minerals (e.g., magnetite), (5) easily oxidizable minerals (e.g., secondary sulfides), (6) resistant oxidizable minerals (e.g., primary sulfides), and (7) residues and silicates. The concentrations of 35 elements in each eluate were analyzed with inductively coupled plasma optical emission spectrometry (ICP-OES) and converted into mg/kg released from the tailings. The detection limit for elements extracted in each step was 10 mg/kg. Total concentrations in the tailings were determined by inductively coupled plasma atomic emission spectroscopy (ICP-AES) and inductively coupled plasma mass spectrometry (ICP-MS) after digestion via sodium peroxides fusion in graphite crucibles.

### Groundwater and surface water sampling

Groundwater in the tailings and surface water downstream of the tailings were sampled monthly during May to October in 2018. Groundwater was pumped using a portable Masterflex® peristaltic pump (Cole-Parmer® International, Chicago, IL, USA) connected to a silicon tube (9 mm) at one pipe (P7) (Fig. 1). The water was pumped for 10–15 min to remove stagnant groundwater before determination of pH, electrical conductivity (EC), and temperature. The O_2_-concentration was measured during sampling in October with an O_2_-meter. To avoid oxygenation of the groundwater, the tube was connected directly to a vacuum Sterifil® Aseptic System and Holder from Merck Millipore with a 42-mm diameter. The water was filtered directly in dark conditions to avoid precipitation of hydrous ferric oxides on the filters.

Surface water was sampled at five sampling points in the catchment area of Smaltjärnen and Morkulltjärnen (designated C7, 11, 13, 14, and 16), as well as a reference point (Ref.) in a neighboring catchment (Fig. 1). Points C7 and C11 were located downstream of Smaltjärnen, while C13 was downstream of Morkulltjärnen. Waters from Smaltjärnen and Morkulltjärnen comingle upstream before reaching sampling points C14 and C16. The surface water was pumped on-line through 142-mm-diameter polycarbonate and acrylic filter holders supplied by Geoteck Environmental Equipment Inc. (Denver, CO, USA). Both groundwater and surface water samples were filtered using 0.22-μm cellulose acetate membrane filters that had been washed with 5% acetic acid for 72 h and rinsed with milliQ water for 24 h (Odman et al. 1999). Screening analyses of 71 elements in the filtered groundwater and surface water (dissolved phase) were carried out by ALS Scandinavia, using inductively coupled plasma sector field mass spectrometry (ICP-SFMS), while sulfate and fluoride were analyzed by ion chromatography (CSN ISO 10304-1, CSN EN 16192). All analyses were carried out in duplicate, with appropriate blanks and standards for quality control. In all cases, the samples were not acidified in the field, which could enhance insoluble tungstic acid formation (Bednar et al. 2010), and the samples were analyzed as soon as possible to prevent W from sticking to the vessel walls (Gustafsson 2003). Particulate phases trapped on the filters used in the surface water filtration were analyzed following the same procedure as the dissolved phase by ALS Scandinavia after lithium metaborate and HNO_3_/HF/HCl digestion.

The filter holders were cleaned with 5% HNO_3_ between each sampling occasion, and blanks were obtained after the cleaning process for controls. Each filter holder was used solely for samples collected from a specific sampling location. New silicon tubes were used each time, and samples were taken from lower to higher concentrations to minimize cross-contamination.

## Results

### Geochemical conditions in P4

**Fig. 2:**
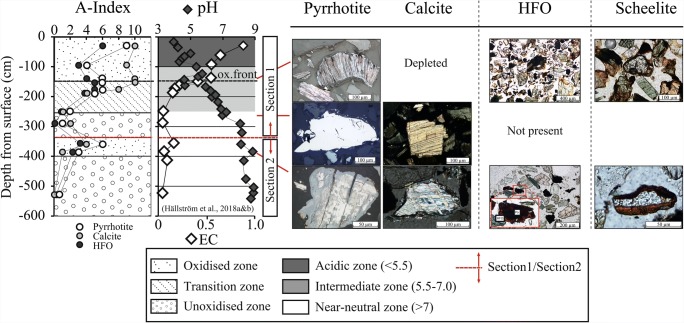
Alteration indices (AI, for pyrrhotite, calcite, and HFO; see illustrative microscopic images, and text for methodological details); pH and EC measurements indicate two distinct sections in the P4 core. Amorphous HFO around scheelite at 1.5 m depth and goethite around scheelite at 3.6 m depth are shown to the right

Two distinct sections were found in the P4 core based on the minerals’ alteration indices and measurements of pH and EC in the tailings, indicating two periods of deposition (Fig. 2). The upper section (section 1) extended from the ground surface down to 3.5 m, and the lower section (section 2) from 3.5 to 6 m.

The upper section contained an oxidized acidic zone (pH < 5.5), a transition zone with intermediate pH (5.5–7), and an unoxidized zone with near-neutral pH (> 7). The oxidized acidic zone occurred between the ground surface and 1.5 m depth. The alteration indices indicated a strongly oxidizing environment in this section, with pyrrhotite completely replaced by HFOs (AI = 9), depleted calcite (AI = 10) and amorphous HFO present, mainly in rims around all grains (AI = 4–6) (Fig. 2). Scheelite grains with a yellow rim were occasionally found at this depth. The transition zone was between 1.5 and 2.5 m depth, with partly oxidized pyrrhotite, partly weathered calcite, and lower abundance of HFO (AI values: 2, 2, and 1 respectively). The unoxidized zone was between 2.5 and 3.5 m, with intact pyrrhotite and calcite, and low abundance of HFO. At this depth, secondary precipitated orthogonal calcite was found frequently (Hällström et al. 2018a).

**Fig. 3:**
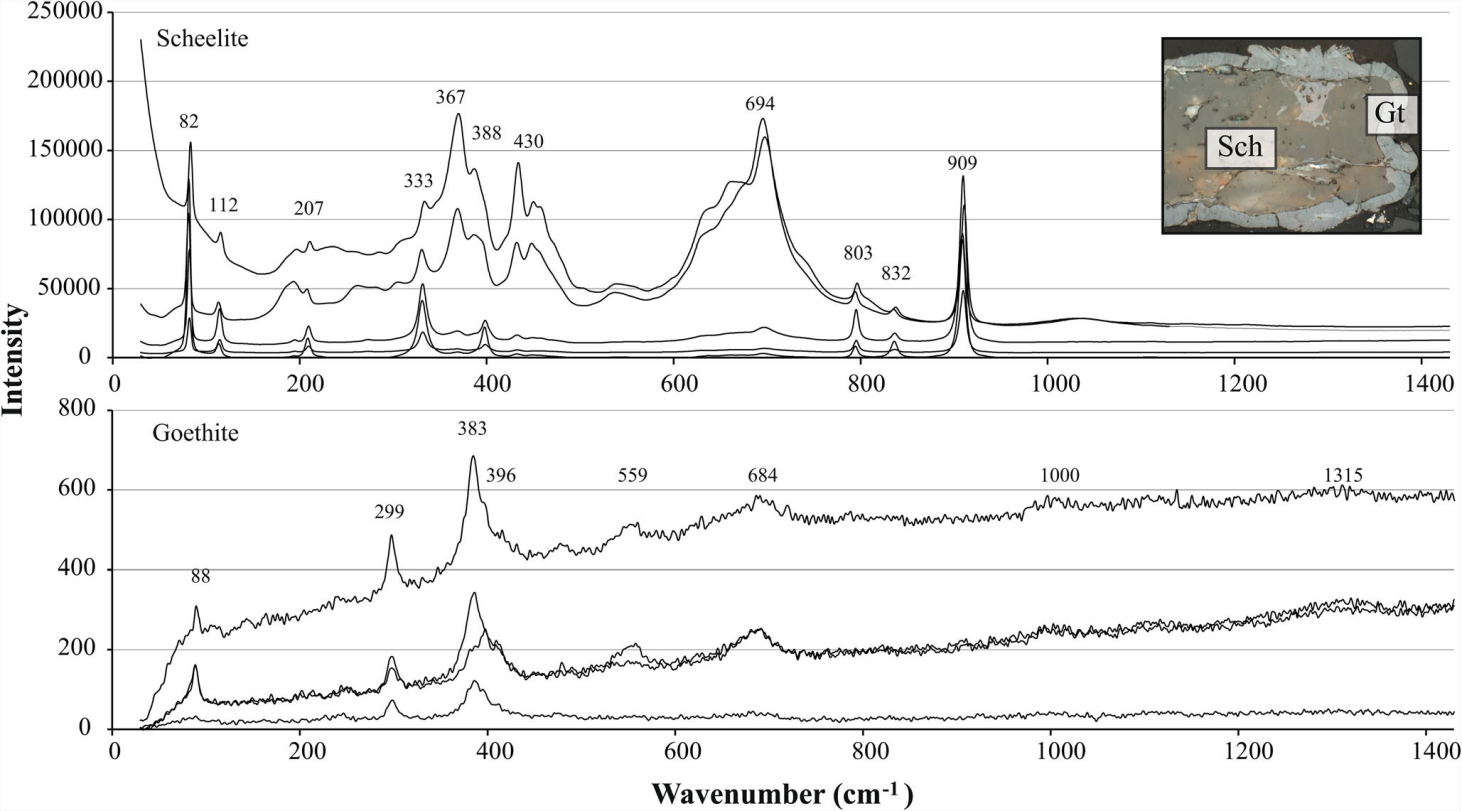
Raman spectra of scheelite with rims of goethite from 3.6 m depth in the tailings

In the deeper tailings (section 2), another oxidized environment was distinguished by the AI values, at 3.6 m depth (Fig. 2). Pyrrhotite and calcite were partly weathered, and crystalline HFO was present as rims on pyrrhotite, magnetite, and scheelite grains (Figs. 2 and 3). Hydrous ferric oxides were not observed to the same extent on larger scheelite grains. The rims of HFO around the smaller scheelite grains were up to 30 μm thick and surrounded parts of or the whole grains. Reflected light optical microscopy and SEM-EDS measurements showed that boundaries between scheelite and HFO were sharp, while those between HFO and pyrrhotite/magnetite were inter-grown. Raman spectroscopic analyses of unweathered scheelite detected clear Raman bands at 82, 112, 207, 333, 367, 388, 430, 694, 803, 832, and 909 cm^−1^ (Fig. 3), in agreement with other scheelite specimens (Rruff 2018). However, limited Raman spectroscopy data of scheelite is available. Raman bands 367 and 694 cm^−1^ were more intense for two of the scheelite grains. The spectroscopically examined HFO had bands at 88, 299, 383, 559, 684, 1000, and 1315 cm^−1^, in agreement with those of goethite (Kreissl et al. 2016).

### 7-step sequential extraction

**Fig. 4.**
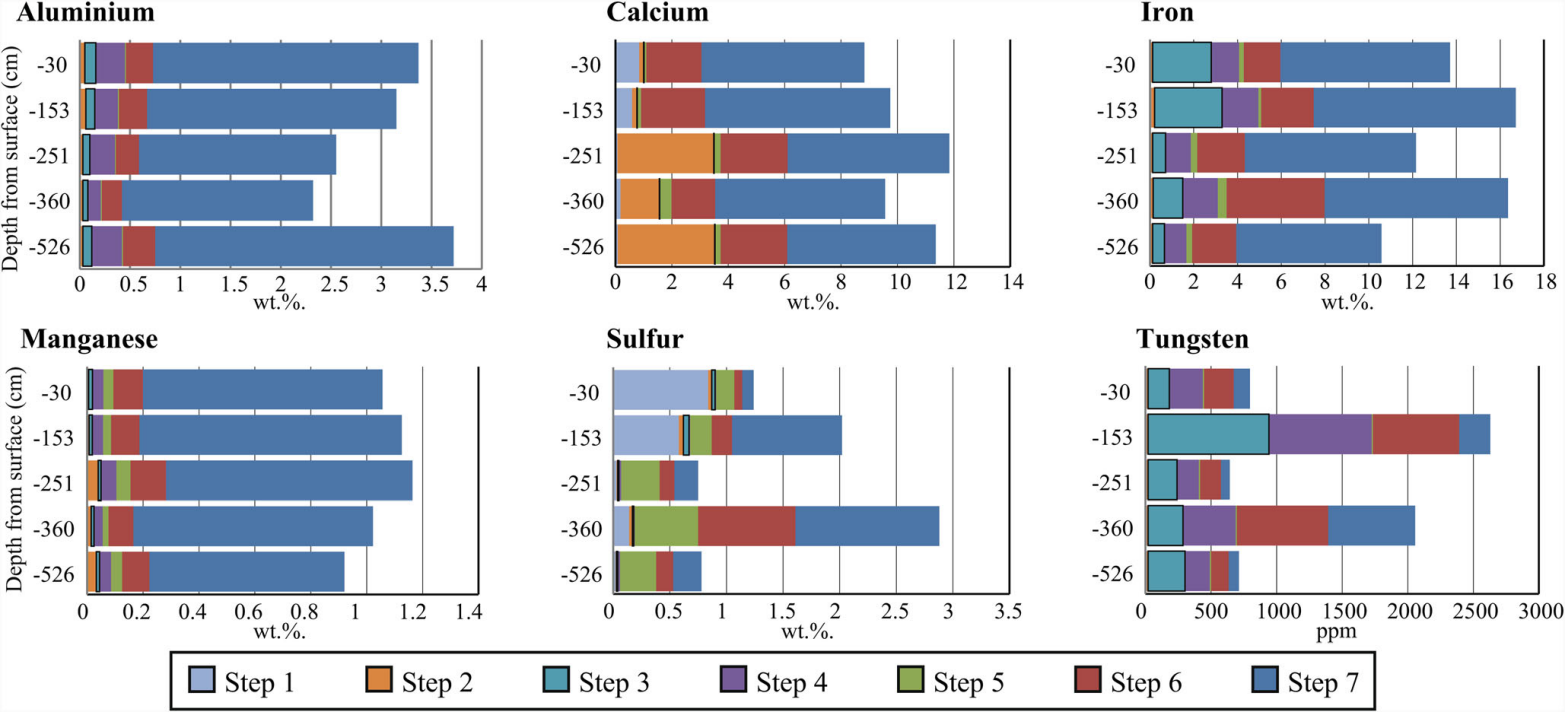
Amounts of Al, Ca, Fe, Mn, S, and W extracted from samples, at indicated depths, of the P4 core in the 7-step sequential extraction process, yielding (step 1) water-soluble phases, (step 2) exchangeable phases, (step 3) easily reducible minerals, (step 4) resistant reducible minerals, (step 5) easily oxidizable minerals, (step 6) resistant oxidizable minerals, and (step 7) residues and silicates

Neither Fe nor W was released in concentrations above the detection limit in the first two steps of the 7-step sequential extraction, but both elements were released from easily reducible phases (step 3) through the whole profile of P4 (Fig. 4). The highest concentration of W released in step 3 was at 1.5 m (922 ppm), and the highest concentrations of Fe were at 0.3 and 1.5 m. Tungsten was released in steps 4, 5, 6, and 7 in various amounts throughout the profile. Iron was released in step 4 (1.3 wt% on average) and step 6 (2.5 wt% on average) originating from magnetite and sulfides. Approximately 60–70% of the total Fe content in the tailings (8.0 wt% on average) was in the residues and silicates obtained in step 7.

High amounts of Ca and S were released in step 1 from 0.3 and 1.5 m depth samples (Fig. 4), with an identical molar ratio to that of gypsum (CaSO_4_). In step 2, up to 4 wt%. of Ca was released from samples below 2.5 m depth, originating from calcite. Iron and W were both released from secondary oxyhydroxides (step 3) throughout the whole profile. The highest concentration of W released in step 3 was at 1.5 m (922 ppm), and the highest concentrations of Fe were at 0.3 and 1.5 m. Aluminum and Mn were mainly found in extracts obtained in steps 4, 6, and 7, indicating that they were in more stable minerals. Approximately 80, 60–70, 60–70, and 70–80% of the total Al, Ca, Fe, and Mn contents in the tailings were in the residues and silicates obtained in step 7, in good agreement with amounts in silicates calculated by a modified element to mineral conversion (EMC) in a previous study by Hällström et al. (2018a).

### Element concentrations in groundwater and surface water

The pH, EC, and O_2_ concentration of the groundwater at P7 was found to be 6.3 ± 0.1 and 2.6 ± 0.06 mS/cm and 6.4 mg/L, respectively, and its level varied between 0.7 and 1.3 m during the sampling occasions (Table 1). Dissolved concentrations of the major elements (Al, Ca, Fe, K, Mg, Mn, Na, S, and Si) were observed in the groundwater with small variations between the sampling occasions. Calcium and S were the dominant elements (average concentrations: 512 and 492 mg/L, respectively). There was larger variation in the concentration of dissolved W (3 μg/L in May, and ≈ 22 μg/L) from June to October.

**Table 1:** pH, EC (μS/cm), concentrations of major elements (mg/L) and W (μg/L) in dissolved (Dis) and particulate (Par) fractions in groundwater of Smaltjärnen Repository (P7), surface water downstream of Smaltjärnen (C7 and C11) and Morkulltjärnen (C13), mixed water of the two surface waters (C14 and C16), and reference samples (Ref.) from May to October 2018. W concentrations in brackets may be contaminated

	Date	pH	EC	O2	Al		Ca		Fe		K		Mg		Mn		Na		S		Si		W	
					*Dis*	*Par*	*Dis*	*Par*	*Dis*	*Par*	*Dis*	*Par*	*Dis*	*Par*	*Dis*	*Par*	*Dis*	*Par*	*Dis*	*Par*	*Dis*	*Par*	*Dis*	*Par*
P7	May	6.5	2500		30		635		37		29		31		44		15		639		17		3	
	Jun	6.2	2670		34		642		57		29		28		42		15		590		18		21	
	Jul	6.2	2590		37		602		56		32		31		41		16		610		21		22	
	Aug	6.2	2640		32		620		52		28		26		37		14		577		17		21	
	Oct	6.2	2640	6	29		575		50		27		24		33		11		535		18		25	
Ref.	May	5.0	18		0.22	0.02	1.52	0.07	0.65	0.27	0.20	0.10	0.39	0.01	0.03	0.01	1.24	0.08	0.51	0.34	2.60	0.01	0.02	0.04
	Jun	6.4	37		0.05	0.08	3.95	0.17	0.35	1.32	0.32	0.11	0.68	0.02	0.48	0.01	1.51	0.14	0.53	0.49	1.53	0.05	0.08	0.04
	Jul	6.6	38		0.06	0.06	4.27	0.19	0.63	0.97	0.45	0.05	0.74	0.02	0.02	0.01	1.74	0.08	0.57	0.28	1.60	0.08	0.02	(0.37)
	Aug	6.3	40		0.06	0.02	4.08	0.13	0.86	0.62	0.15	0.06	0.67	0.01	0.02	0.01	1.39	0.09	0.62	0.30	1.66	0.04	(0.52)	0.09
	Sep	6.5	35		0.10	0.03	4.09	0.15	0.74	2.00	0.28	0.09	0.77	0.01	0.01	0.01	1.67	0.04	0.77	0.19	1.93	0.07	0.01	0.21
	Oct	6.5	38	11	0.15	0.02	4.17	0.08	1.24	0.33	0.33	0.05	0.69	0.01	0.02	0.01	1.46	0.06	0.84	0.23	1.91	0.05	0.07	0.01
C7	May	5.7	100		0.22	0.02	12.4	0.10	0.77	0.32	0.80	0.12	0.93	0.01	0.26	0.01	2.05	0.07	11.76	0.32	2.57	0.01	0.17	0.26
	Jun	5.8	256		0.23	0.07	43.3	0.36	2.13	2.63	1.52	0.25	2.36	0.02	1.91	0.02	2.84	0.11	40.20	0.66	2.57	0.09	0.14	0.66
	Jul	5.2	306		0.23	0.08	41.9	0.31	2.81	2.94	1.96	0.06	2.72	0.02	1.18	0.01	3.14	0.04	43.56	0.38	3.40	3.08	0.10	(0.75)
	Aug	5.5	460		0.27	0.03	65.6	0.33	3.12	2.83	2.83	0.10	3.93	0.02	1.71	0.02	3.44	0.05	66.50	0.50	3.16	0.49	(0.62)	0.25
	Sep	5.9	555		0.26	0.07	94.4	0.64	2.46	4.65	3.66	0.52	5.92	0.04	2.19	0.02	4.86	0.16	91.21	1.14	4.56	0.50	0.02	1.04
	Oct	6.2	396	11	0.16	0.11	52.6	0.59	1.21	3.36	2.31	0.25	3.01	0.03	1.11	0.01	3.26	0.13	49.03	0.80	3.70	0.19	0.07	0.17
C11	May	5.6	7		0.24	0.02	8.95	0.07	1.14	0.22	0.67	0.07	0.75	0.01	0.17	0.01	1.82	0.05	8.09	0.21	2.48	0.01	0.23	0.17
	Jun	5.7	163		0.21	0.06	21.6	0.21	0.90	1.41	0.89	0.18	1.54	0.02	0.64	0.01	2.19	0.09	18.54	0.45	2.53	0.08	0.14	0.38
	Jul	5.8	259		0.25	0.05	36.1	0.27	0.95	1.49	2.09	0.07	2.50	0.02	0.50	0.01	3.09	0.06	36.10	0.33	3.34	1.38	0.09	(0.23)
	Aug	5.8	402		0.35	0.02	55.8	0.28	0.69	1.77	2.54	0.12	3.44	0.02	1.32	0.01	3.27	0.05	55.37	0.46	3.00	0.08	(0.60)	0.13
	Sep	6.0	472		0.28	0.03	74.7	0.06	1.59	0.45	2.82	0.04	5.06	0.01	1.73	0.01	4.33	0.04	74.54	0.13	4.39	0.08	0.03	0.59
	Oct	6.4	322	12	0.17	0.10	42.5	0.45	1.51	2.37	1.77	0.20	2.61	0.03	0.81	0.01	2.93	0.10	39.79	0.64	3.56	0.07	0.12	0.12
C13	May	6.1	27		0.11	0.04	2.52	0.10	0.32	0.54	0.33	0.09	0.37	0.01	0.05	0.01	1.75	0.09	0.91	0.33	2.60	0.08	0.38	0.64
	Jun	6.5	27		0.08	0.03	2.86	0.07	0.51	0.30	0.30	0.08	0.38	0.01	1.59	0.01	1.57	0.07	0.83	0.31	2.11	0.08	0.85	0.38
	Jul	7.6	29		0.08	0.03	2.87	0.11	0.58	0.27	0.51	0.04	0.41	0.01	0.01	0.01	1.88	0.06	0.81	0.22	2.23	0.26	1.06	(1.09)
	Aug	6.7	29		0.08	0.02	2.92	0.12	0.72	0.36	0.29	0.06	0.39	0.01	0.03	0.02	1.57	0.08	0.75	0.32	1.68	0.17	(1.72)	0.55
	Sep	6.6	30		0.06	0.07	3.20	0.27	0.62	0.88	0.38	0.35	0.48	0.03	0.03	0.01	1.99	0.35	0.98	1.00	2.46	0.17	1.02	0.20
	Oct	6.6	30	11	0.07	0.06	2.87	0.11	1.11	0.58	0.28	0.06	0.39	0.02	0.02	0.05	1.54	0.06	0.78	0.22	2.07	0.06	1.74	1.31
C14	Jun	6.6	66		0.09	0.04	7.83	0.11	0.57	0.65	0.48	0.11	0.74	0.01	1.66	0.02	1.75	0.08	5.46	0.34	2.24	0.05	0.57	0.53
	Jul	6.4	63		0.07	0.02	7.15	0.08	0.59	0.32	0.66	0.02	0.76	0.01	0.04	0.03	2.05	0.03	5.05	0.12	2.47	0.15	0.83	(0.60)
	Aug	6.3	107		0.05	0.02	12.9	0.09	0.37	0.36	0.77	0.05	1.17	0.01	0.05	0.03	2.05	0.05	9.82	0.21	2.11	0.04	(0.98)	0.88
	Oct	6.2	8	11	0.10	0.02	11.4	0.10	0.89	0.50	0.45	0.07	0.94	0.01	0.16	0.01	1.82	0.06	8.87	0.26	2.43	0.04	1.07	0.32
C16	Jun	6.5	37		0.08	0.04	4.00	0.08	0.59	0.50	0.25	0.08	0.50	0.01	0.13	0.03	1.63	0.07	1.77	0.28	2.22	0.04	0.92	0.61

In the surface water downstream of Smaltjärnen (at points C7 and C11), the average pH and EC were 5.8 and 300 μS/cm, respectively, while the pH and EC of the reference, C13, C14, and C16 samples were > 6 and < 40 μS/cm, respectively. Dissolved concentrations of Al, Ca, Fe, K, Mg, Mn, Na, S, and Si in C7 and C11 samples were all high (0.2, 45, 1.6, 2.0, 2.9, 1.1, 3.1, 44, 3.2 mg/L on average, respectively) compared with reference samples (0.1, 3.7, 0.7, 0.3, 0.7, 0.1, 1.5, 0.6, and 1.9 mg/L, respectively) (Table 1). The concentrations were lowest in May and increased gradually until September, coinciding with decreased waterflow. All the major elements, except Fe, were mainly present in the dissolved phase (95%, on average) on all sampling occasions in C7 and C11 samples. The percentage of Fe present in the dissolved phase varied between 16 and 73%, with an average of 51%. The concentrations of particulate Fe decreased between C7 and C11, with the C7/C11-ratio: 1.5, 1.9, 2.0, 10.3, and 1.4 in May, June, July, August, September, and October, respectively.

**Fig. 5:**
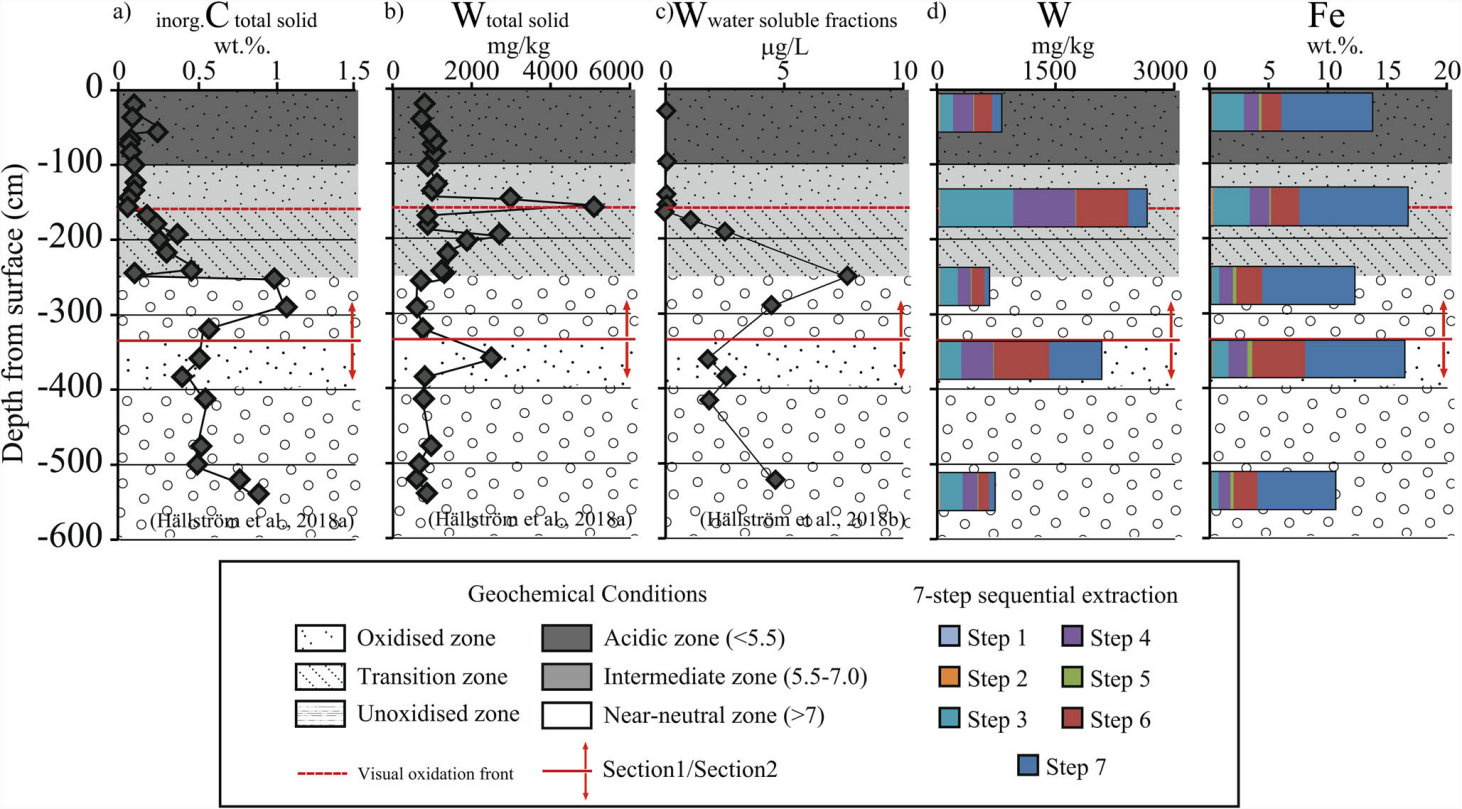
Results of analysis of the P4 core. (a) Total inorganic C content (wt%), (b) total W content (mg/kg), (c) W concentration in water-soluble phases, and (d) W and Fe contents in fractions obtained from the 7-step sequential extraction: (1) water-soluble phases, (2) exchangeable phases, (3) easily reducible minerals, (4) resistant reducible minerals, (5) easily oxidizable minerals, (6) resistant oxidizable minerals, and (7) residues and silicates

Total detected W concentrations were higher in surface waters at all the sampling points than in the reference samples. Tungsten was present mainly in the particulate phase (65% on average) at C7 and C11. The maximum total W concentration there was 1 μg/L, 98% of which was in the particulate phase. The concentrations of particulate W decreased between C7 and C11, with the C7/C11-ratio: 1.5, 1.7, 3.3, 1.9, 1.8, and 1.4 in May, June, July, August, September, and October, respectively. Dissolved W concentrations at C7 and C11 were not elevated compared with the reference samples (< 0.2 μg/L). The highest detected concentrations of W in both dissolved and particulate phases were at C13 (1.7 and 1.3 μg/L, respectively). The total concentrations of W at C14 and C16 were lower than at C13 but significantly higher than at C11. The concentrations of dissolved and particulate W in all reference samples were < 0.09 μg/L, except for in one dissolved sample (August) and one particulate sample (July). The elevated concentrations could have been due to contamination of the sampling equipment, an analytical error, or naturally occurring W in the water. Measurements from these occasions are placed in brackets in Table 1 and omitted in Fig. 5. No other element was found in abnormal concentrations in the reference sample, and the contamination is therefore assumed to only include W. No elevated concentrations of W were detected in blanks obtained after cleaning the filter equipment.

## Discussion

Tungsten was primarily found in scheelite (CaWO_4_) with an abundance of 0.1 wt% in the tailings at Yxsjöberg. Scheelite is considered to be a relatively stable mineral (Bokii and Anikin 1956), but little attention has been paid to its stability and the geochemical behavior of W in tailings. Scheelite grains with three characteristics were found in the tailings by optical microscopy: (1) unaltered grains, (2) grains with yellow rims at 1.5 m depth, and (3) grains with HFO rims at 3.6 m depth. Accordingly, variations in W concentration with depth have been previously detected at P4 and P7, including clear peaks at 1.5 and 3.6 m depths at P4 (Hällström et al. 2018a) (Fig. 5).

### Section 1: W mobility in the later deposition period

Detailed analysis of the P4 core using the 7-step sequential extraction procedure showed that 30% of W (922 mg/kg) in the peak at 1.5 m was associated with easily reducible phases (Fig. 5). However, the extraction procedure was not developed for scheelite and some uncertainties and analytical problems were encountered. For example, it was not clear in which step scheelite dissolved and amounts of W extracted in the seven steps are substantially smaller than total amounts detected in the tailings. Old scheelite concentrate from the processing plant was analyzed by the sequential extraction in attempt to clarify in which step scheelite was dissolved, without success. With that in mind, the 7-step sequential extraction results indicated that weathering of scheelite and secondary capturing of W in easily reducible phases had occurred in the upper-parts of the tailings where pH was > 7. Concentrations of W in the water-soluble (Hällström et al. 2018b) and exchangeable phases were below the detection limit, indicating that W was strongly bound to the easily reducible phases. Indications of scheelite alteration were observed by optical microscopy as formations of a yellow rim inter-grown on scheelite surfaces, as reportedly formed through incongruent weathering of scheelite with precipitates of insoluble tungstic acid (H_2_WO_4_) (Marinakis and Kelsall 1987; Montgomery and McKibben 2012). According to Montogmery and McKibben (Montgomery and McKibben 2012), tungstic acid forms during scheelite dissolution at pH 3 or lower; above this pH, the rate of scheelite dissolution increases with pH and temperature.

Iron was the dominating oxyhydroxide at this depth and adsorption/co-precipitation with HFO has been proven to immobilize W at pH conditions below 8 (Gustafsson 2003). This can occur by either W substituting Fe^3+^ in the crystal lattice of ferrihydrite (Kreissl et al. 2016) or formation of strong inner-sphere complexes (Kashiwabara et al. 2013). In the upper tailings (at depths < 1.5 m), amorphous HFO was present around all examined mineral grains, it had replaced pyrrhotite completely, and formed thicker rims around chalcopyrite and pyrite. Iron originated mainly from oxidation of pyrrhotite (Eqs. 1 and 2) (Hällström et al. 2018a).

1 $$ {\mathrm{Fe}}_{\left(1-x\right)}\mathrm{S}+\left(2-\frac{x}{2}\right){\mathrm{O}}_2+x{\mathrm{H}}_2\mathrm{O}\leftrightarrow \left(1-x\right){\mathrm{Fe}}^{2+}+{\mathrm{SO}}_4^{2-}+2x{\mathrm{H}}^{+}\left(x=0.08\right) $$

2 $$ {\mathrm{Fe}}^{2+}+\frac{1}{4}{\mathrm{O}}_2+\frac{5}{2}{\mathrm{H}}_2\mathrm{O}\leftrightarrow \mathrm{Fe}{\left(\mathrm{OH}\right)}_{3(s)}+2{H}^{+} $$

It was not possible to distinguish the type of HFO present in the oxidized acidic tailings due to the amorphous structure, but from the literature, 2-line ferrihydrite is known to be the first phase formed during early stages of Fe^2+^ oxidation at low temperatures under near-surface conditions with pH > 4 (Kreissl et al. 2016). Except for HFO, Al and Mn oxyhydroxides have also been seen to scavenge W (Bauer et al. 2017a; Hur and Reeder 2016). However, Al and Mn contents of fractions obtained in step 3 in the extraction of samples from Yxsjöberg were much lower than the Fe contents, so the adsorption/co-precipitation of W to their oxyhydroxides is assumed to be insignificant.

**Fig. 6:**
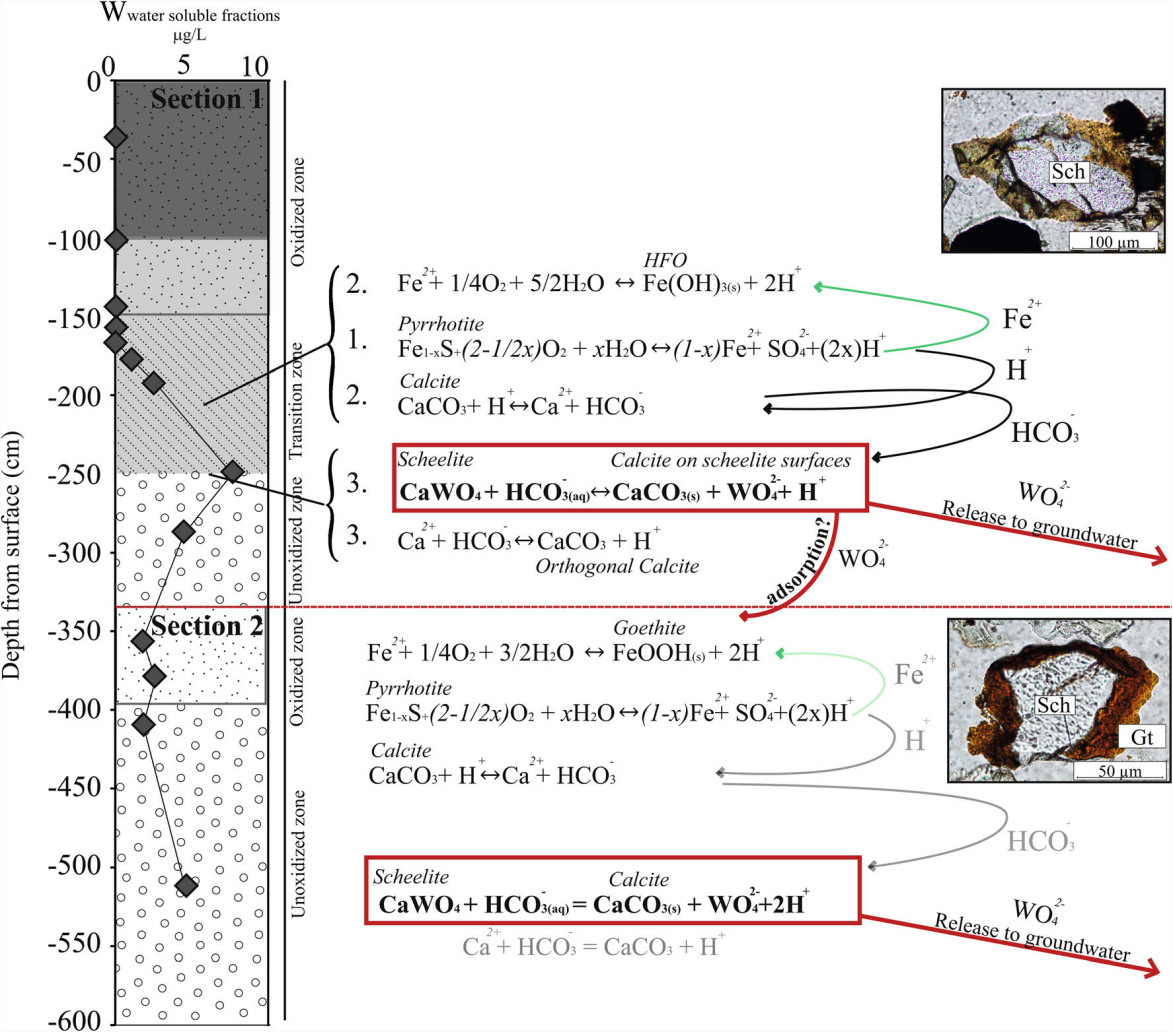
The hypothesized sequence of (1) pyrrhotite oxidation ➔ (2) calcite depletion ➔ release of HCO_3_^2−^ and transport downwards ➔ (3) anion exchange between HCO_3_^2−^ and WO_4_^2−^ ➔ WO_4_^2−^ release to groundwater or adsorption to goethite, illustrated with depth in sections 1 and 2. Microscopic images of altered scheelite at 1.5 m and scheelite with goethite rim at 2.6 m are shown in the pictures

At present time, the release of W was ongoing at 2.5 m depth in unoxidized tailings with near-neutral pH (Fig. 5), and associated reactions putatively involved (across the profile in the tailings) are illustrated in Fig. 6. At 2.5 m depth, elevated concentrations of W were released in water-soluble phases correlated with lower content of W in the tailings. At the same depth, the pH is above 7 and C has precipitated mainly as secondary orthogonal calcite (Hällström et al. 2018a). Water-soluble phases of W coinciding with accumulation of C have also been found in another core from Yxsjöberg (Salifu et al. 2018). Siderite (FeCO_3_) is commonly reported in secondary carbonates in tailings from sulfidic deposits (Blowes et al. 2003), but was not found in the thin section from 2.5 m depth. The high abundance of Ca released from fluorite and calcite weathering could favor formation of secondary Ca-carbonates rather than Fe-carbonates. The formation of secondary carbonates is supported by enriched C-isotope ratios at similar depths in the additional core from the Yxsjöberg tailings (Salifu et al., unpublished data). The accumulated C was originally released in the upper acidic oxidized and transition zones due to calcite neutralizing acid produced from pyrrhotite oxidation (Eq.3) and transported downwards to conditions where pH > 7.

3 $$ \mathrm{CaC}{\mathrm{O}}_3+{\mathrm{H}}^{+}\leftrightarrow {\mathrm{Ca}}^{2+}+{\mathrm{H}\mathrm{CO}}_3^{-} $$

It is hypothesized that small proportions of CO_3_^2−^ have displaced WO_4_^2−^ at the surfaces of scheelite at this depth, instead of precipitating as calcite, thereby releasing W to the pore water according to Eq. 4, and as illustrated in Fig. 4.

4 $$ \mathrm{Ca}{\mathrm{WO}}_{4(s)}+{\mathrm{H}\mathrm{CO}}_{3\ (aq)}^{-}\leftrightarrow {\mathrm{CaCO}}_{3(s)}+{\mathrm{WO}}_4^{2-}+{\mathrm{H}}^{+} $$

This has not been mineralogically confirmed, due to the lack of scheelite in the thin section from this depth, but is in good agreement with results presented by Atademir et al. (1979), Marinakis and Kelsall (1987), and Montgomery and McKibben (2012). At Yxsjöberg, elevated concentrations of dissolved W were found in the groundwater of the tailings (up to 22 μg/L) and elevated concentrations of total W in the surface water downstream of the repository (up to 1 μg/L) compared with the reference water (< 0.2 μg/L). These findings confirm that W is released from scheelite in the tailings and transported out of the impoundment. Weathering of scheelite in the tailings is assumed to be an indirect effect of the pyrrhotite oxidation. Hence, skarn tailings without sulfide oxidation or without contact with atmosphere or limited amount of calcite might produce mine water drainage with limited concentrations of W.

### Section 2: W mobility in the older deposition period

A similar sequence to the one inferred in section 1 with pyrrhotite oxidation → calcite alteration → downward transport of CO_3_^2−^, → accumulation of C → decrease solid W and increase water-soluble phases of W occurred in section 2 (Fig. 4). Compared with section 1, the HFO formed in the older oxidized zone in section 2 with near-neutral pH were crystalline goethite, and were only present around pyrrhotite, magnetite, and scheelite (Figs. 2 and 6). The occurrence of goethite as rims on scheelite has been studied in the laboratory by Gao et al. (2016) and is due to negatively charged surfaces of scheelite attracting Fe^3+^. Low concentrations of W are released into the water-soluble phase at this depth (Hällström et al. 2018b). The rims could inhibit scheelite weathering by limiting the possibility of CO_3_^2−^ released from upper layers interacting with the scheelite surfaces. Released WO_4_^2−^ could also be scavenged in the tailings by adsorption or co-precipitation processes (Kreissl et al. 2016), which would reduce concentrations of W in the water-soluble phases and reaching the groundwater. Tungstate currently released from the tailings lying above in section 1 would adsorb to the already existing goethite, and WO_4_^2−^ released during primary formation of HFO could be co-precipitated in the crystal lattice of goethite (Kreissl et al. 2016).

### W mobility in groundwater and surface water

**Fig. 7:**
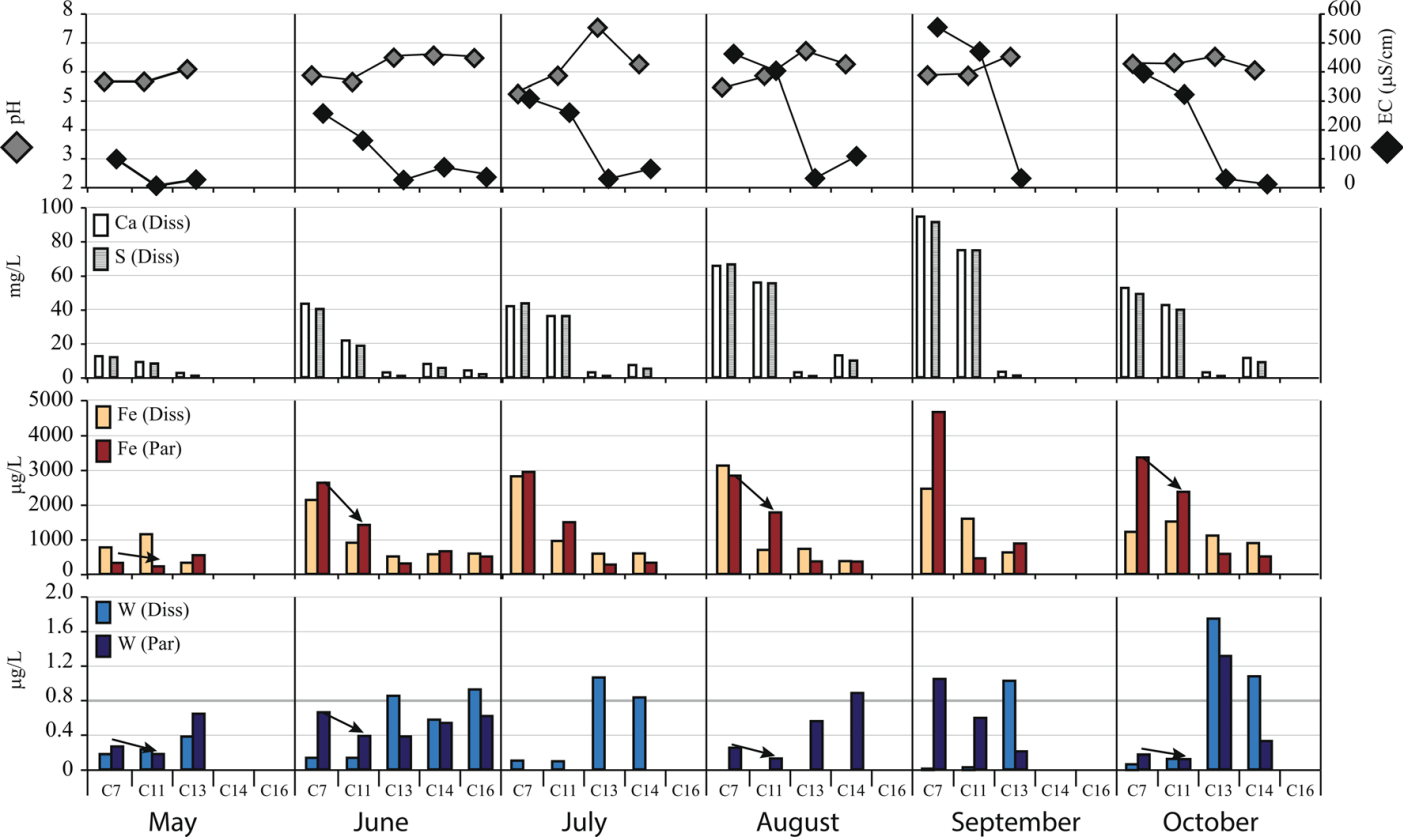
Monthly pH, EC, dissolved (Diss) Ca and S, dissolved and particulate (Par) Fe, and dissolved and particulate W at C7, C11, C13, C14, and C16. The arrows indicate times when the sedimentation ratio of Fe between C7 and C11 was similar to that of W

The long-term storage of tailings in ambient conditions with apparent pyrrhotite oxidation, calcite depletion, silicate, and dissolution of secondary gypsum has affected the water quality downstream of the Smaltjärnen Repository. A higher rate of pyrrhotite oxidation than calcite neutralization in the tailings has resulted in release of acid to the surface water, generating low pH at C7 and C11 (Fig. 7), although neutralization calculations showed that calcite should have the potential to neutralize the acidity produced (Hällström et al. 2018a). The higher rate of pyrrhotite weathering compared with calcite shows the necessity of other prediction tools than only static tests. The EC was ≈ 345 μS/cm downstream Smaltjärnen Lake (C7), indicating a high release of ions into the water. Calcium and S were the dominating elements in the surface water, with concentrations up to 94 and 91 mg/L (273 mg/L SO_4_^2−^), respectively, and they were nearly entirely present in the dissolved phase (> 99%) downstream of the repository (C7 and C11). The concentrations were lower during the spring flood in May and increased gradually with reductions in water flow until September (Fig. 7). The molar Ca to S ratio was close to that of gypsum (1:1.2–1.3, molar ratio of Ca:SO_4_), showing that some of the secondary gypsum formed in the upper oxidized acidic tailings has been dissolved and transported from the repository. The concentrations of released Ca and SO_4_^2−^ are relatively low compared with those detected in other W mining areas (Candeias et al. 2015) and the SO_4_^2−^ concentration was below the threshold value for taste in drinking water (370 mg/L) (World Health Organization 2004).

Iron was present in both dissolved and particulate phases in the surface water downstream of Smaltjärnen, and the highest concentrations were detected at C7 on all sampling occasions. There, Fe was the main particulate element, accounting for 53%, on average, of all elements in fractions > 0.2 μm. Total concentrations of Fe released from the tailings were significantly lower than the S concentrations, indicating that a large proportion of Fe released from pyrrhotite oxidation had been captured as HFO in the tailings. This is consistent with results of the 7-step sequential extraction. The Fe concentrations at C11 were similar to those in the reference samples, suggesting that large proportions of Fe precipitated in the sediments between C7 and C11. The Fe concentrations at C13, C14, and C16 were similar to the reference values (Fig. 7).

The concentrations of W in the groundwater from Smaltjärnen Repository and downstream surface waters were low on all sampling occasions, but still higher than in the reference sample. Tungsten was mainly present in the particulate fraction at sampling points C7 and C11, coinciding with the high concentrations of particulate Fe. Particulate W is known to have high affinity for particulate Fe in natural rivers (Bauer et al. 2017b) and a correlation between the sedimentation of Fe and W between C7 to C11 was found. Thus, it is hypothesized that tungsten was released as WO_4_^2−^ from the groundwater in the Smaltjärnen tailings and adsorbed to particulate Fe precipitated in the surface water. The concentrations of W and Fe in groundwater (at P7) were approximately 30 times higher than those at C7 during May, and decreased similarly with decreasing water flow in the surface water. The difference may be due to either (co-)precipitation of secondary minerals or dilution by the water in Smaltjärnen Lake. However, the differences in concentrations were of the same order of magnitude as the drop in Mg concentration (10- to 30-fold) between P7 and C7. Magnesium is a good dilution indicator because it does not readily enter secondary minerals or biota. Thus, these findings suggest that most Fe and W leaving the repository with the mine drainage are transported away from the repository by the surface water. Particulate W is hypothesized to subsequently settle between C7 and C11, with particulate Fe, thus, the ratio between W in C7 and C11 and Fe was similar in May, June, August, and October.

No environmental regulations or general guideline values for dissolved W in surface waters have been set in the EU or USA, due to the lack of knowledge of its mobility and toxicity (Koutsospyros et al. 2006; Strigul 2010). In Russia, a maximum allowed concentration of 0.8 μg/L dissolved W has been set for aquatic systems used for fishing (Strigul et al. 2009). The concentrations of W in the dissolved phase downstream of Smaltjärnen were all below this threshold, and thus too low for it to be considered hazardous according to this guideline. However, dissolved concentrations of W above 0.8 μg/L were detected in surface water downstream of Morkulltjärnen Repository (at C13) during June to October. The pH at C13 varied between 6.0 and 7.6, and low concentrations of particulate Fe were present. Thus, monometric tungstate was probably the dominating species (Koutsospyros et al. 2006). The W concentrations in mixed water between Smaltjärnen and Morkulltjärnen at C14 and C16 were highly affected by the high concentrations of W from C13 and concentrations above 0.8 μg/L were measured during June, July, August, September, and October. The W concentrations at C13, C14, and C16 were still low compared with concentrations reported downstream of other W mining areas by Candeias et al. (2015), Gurbanov et al. (2015), and Seiler et al. (2005), but higher than concentrations detected in rivers downstream of industrial areas, e.g., in Japan (Koutsospyros et al. 2006). This indicates that the W contamination previously detected by the municipality originates from Morkulltjärnen Repository. Further investigations, including geochemical characterization of Morkulltjärnen Repository, are needed to understand the release of W from this source.

## Conclusion

- Partial dissolution of scheelite releases some W to mine drainage. The weathering process is hypothesized to be due to anion exchange with CO_3_^2−^ on the surfaces of scheelite in unoxidized conditions where pH is above 7. The CO_3_^2−^ was released during calcite neutralization of acidity produced from pyrrhotite oxidation and thereafter transported downwards in the tailings.
- Some of the W that is released from scheelite can adsorb to HFO and remain immobilized within the tailings. Some of the W that is transported away from the tailings appears to co-precipitate downstream with HFO.
- Elevated concentrations of W were found in the groundwater of the tailings and in surface water downstream the tailings confirming weathering of scheelite. However, the concentrations were not large enough to be classified as a contaminant according to today’s water regulations.
- Released W from scheelite was co-precipitated with amorphous HFO in oxidized acidic tailings, and possibly adsorbed to goethite in an old oxidized layer in the deep tailings.
